# Usability of human Infinium MethylationEPIC BeadChip for mouse DNA methylation studies

**DOI:** 10.1186/s12859-017-1870-y

**Published:** 2017-11-15

**Authors:** Maria Needhamsen, Ewoud Ewing, Harald Lund, David Gomez-Cabrero, Robert Adam Harris, Lara Kular, Maja Jagodic

**Affiliations:** 1 Department of Clinical Neuroscience, Center for Molecular Medicine, Karolinska Institutet, Stockholm, Sweden; 2 Applied Immunology and Immunotherapy, Department of Clinical Neuroscience, Karolinska Institutet Center for Molecular Medicine, Karolinska Hospital at Solna, Stockholm, Sweden; 3 Unit of Computational Medicine, Department of Medicine, Solna, Center for Molecular Medicine, Karolinska Institutet, Stockholm, Sweden; 4 Mucosal and Salivary Biology Division, King’s College London, Dental Institute, London, UK

**Keywords:** Epigenetics, DNA methylation, Infinium BeadChip, Epic, Mouse, Rat, Guinea pig, Rabbit, Sheep, Pig, Cown, Dog, Cat, Macaque, Chimpanzee

## Abstract

**Background:**

The advent of array-based genome-wide DNA methylation methods has enabled quantitative measurement of single CpG methylation status at relatively low cost and sample input. Whereas the use of Infinium Human Methylation BeadChips has shown great utility in clinical studies, no equivalent tool is available for rodent animal samples. We examined the feasibility of using the new Infinium MethylationEPIC BeadChip for studying DNA methylation in mouse.

**Results:**

In silico, we identified 19,420 EPIC probes (referred as mEPIC probes), which align with a unique best alignment score to the bisulfite converted reference mouse genome mm10. Further annotation revealed that 85% of mEPIC probes overlapped with mm10.refSeq genes at different genomic features including promoters (TSS1500 and TSS200), 1st exons, 5′UTRs, 3′UTRs, CpG islands, shores, shelves, open seas and FANTOM5 enhancers. Hybridization of mouse samples to Infinium Human MethylationEPIC BeadChips showed successful measurement of mEPIC probes and reproducibility between inter-array biological replicates. Finally, we demonstrated the utility of mEPIC probes for data exploration such as hierarchical clustering.

**Conclusions:**

Given the absence of cost and labor convenient genome-wide technologies in the murine system, our findings show that the Infinium MethylationEPIC BeadChip platform is suitable for investigation of the mouse methylome. Furthermore, we provide the “mEPICmanifest” with genomic features, available to users of Infinium Human MethylationEPIC arrays for mouse samples.

**Electronic supplementary material:**

The online version of this article (10.1186/s12859-017-1870-y) contains supplementary material, which is available to authorized users.

## Background

Epigenetic mechanisms refer to dynamic processes that integrate internal and external signals and regulate gene expression in a spatiotemporal manner. DNA methylation is the most studied epigenetic mechanism and involves the covalent addition of a methyl group to cytosine primarily in the context of CpG dinucleotides. DNA methylation is established by the de novo methyltransferases, DNMT3A and DNMT3B, and is maintained during cellular division by DNMT1, thereby assuring propagation of the methylation patterns [1]. The cell-type specific methylomes (together with other chromatin modifications such as histone post-translational modifications), lead to unique transcriptional profiles and thereby specific cellular phenotypes. DNA methylation is a stable and heritable mechanism that can persist through cell divisions even in the absence of the original stimuli [2]. Finally, it can be reliably measured from a small amount of input material. These features make DNA methylation an ideal readout of genome activity in various clinical and experimental samples.

The recent development and optimization of methods for quantification of DNA methylation genome-wide have mainly focused on human genomic DNA, with special interest in reducing the sample input while improving both accuracy and coverage. Genome-wide DNA methylation arrays allow absolute measurement of single CpG methylation status located at various regulatory regions throughout the human genome at relatively low cost, thereby giving great utility in clinical studies. The use of Infinium HumanMethylation450 BeadChips (HM450) in epigenome-wide association studies in large cohorts has proven a very promising approach in deciphering putative pathogenic mechanisms influenced by both genetic and environmental factors [3, 4]. However, such tools are failing to explore DNA methylation in animal models, such as in mouse, leaving as the only option labor-consuming and costly methodologies that often require more advanced bioinformatics resources.

Prior investigation of the feasibility of using the HM450 platform on non-human primate and mouse genomes has revealed common probes mapping to bisulfite converted reference genomes [5, 6]. The new Infinium MethylationEPIC BeadChips contains over 850,000 probes, which cover more than 90% of the sites on the HM450, plus more than 350,000 novel CpGs at regions identified as potential enhancers in the FANTOM5 project [7]. Herein we aimed to determine the utility of the new Infinium Human EPIC BeadChip array for studying DNA methylation in mouse.

## Results and discussion

### Mapping of EPIC probes to the mouse genome

**Fig. 1:**
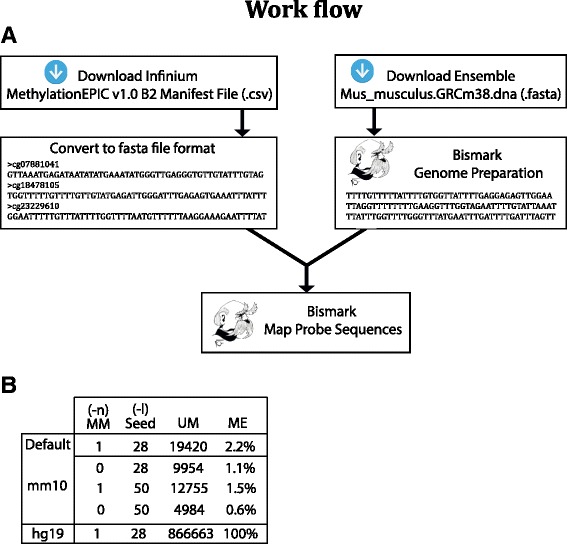
Mapping of EPIC probes to mouse and human genomes. **a** Strategy for mapping EPIC probes to the mouse genome **b** Alignment results of EPIC probes mapped to mouse and human genomes. MM (−n): Mismatch allowance, UM: Uniquely Mapped, ME: Mapping Efficiency and -l: Seed length

To identify EPIC probes with the potential of detecting DNA methylation in mouse, we first pursued an in silico approach whereby probe sequences were mapped to the mouse genome. The most recent genome of the widely used C57BL/6 mouse strain, referred to as mm10 (or GRCm38), was downloaded from Ensembl and used as a reference genome (Fig. 1a). EPIC probe sequences were downloaded from the Illumina website and subsequently converted to the fasta format (Fig. 1a). Importantly, probes from DNA methylation arrays predominantly contain 3 bases (A, T and C) since they are designed to hybridize with genomic DNA, which undergoes sodium bisulfite (BS)-treatment. BS treatment converts unmethylated cytosines into uracils, which during the whole genome amplification step are read as thymines, while methylated cytosines remain unaffected by the BS conversion and are read as cytosines. To account for BS-treatment-dependent reduced complexity of EPIC probes we used the flexible aligner Bismark, which simulates bisulfite conversion of the reference genome in silico prior to mapping (referred to as “Genome preparation”, Fig. 1a) [8]. Other short read alignment tools designed for mapping of bisulfite converted DNA and using slightly different mapping strategies such as Bmap [9] or Novoalign Bisulfite Mode (http://www.novocraft.com) could also be considered. However, comparison of these three alignment tools for former versions of DNA methylation arrays (HM27 and HM450) has demonstrated Bismark to have the largest percentage of uniquely mapped probes overlapping between the three alignment tools [5]. Bismark was thus selected as the primary aligner for EPIC probes.

Bismark relies on Bowtie for mapping, which by default uses a 5′-end seed of 28 nucleotides to initiate the alignment process [10]. Importantly, the methylation site of interest is located at the 3′-end of EPIC probes and hence sequences were processed to their reverse/complements prior to mapping (Fig. 1a). Reverse/complemented EPIC probe sequences are available in Additional file 1 in a fasta file format usable for other strain/species applications. For mapping, default parameters with a seed length of 28 (−l 28) and 1 mismatch (−n 1), as previously used [5], were first tested. We identified 19,420 hits with a unique best alignment score (excludes hits with the same number of mismatches and alignments scores), corresponding to a mapping efficiency of 2.2% (Fig. 1b). As expected, applying more stringent parameters such as a longer seed length of 50 nucleotides (−l 50) and 0 mismatch allowance (−n 0) reduced the number of unique hits and the corresponding mapping efficiency (Fig. 1b). Since DNA methylation arrays are known to accurately hybridize human DNA, despite mismatches caused by widespread genetic variation, the default settings, which allow for 1 mismatch in the seed, were considered acceptable and used in the analysis.

In order to verify our mapping strategy EPIC probes were aligned to the human genome (hg19/GRCh37) using Bismark with default setting (−l 28 and -n 1), which returned 866,663 uniquely mapped hits corresponding to a mapping efficiency of 100% (Fig. 1b).

We thus identified 19,420 EPIC probes (listed in Additional file 2) in silico which align as unique best hits to the mouse genome. For the aim of concision of nomenclature, these probes are referred to as *mEPIC probes* in the rest of the manuscript.

### Annotation of mEPIC probes

**Fig. 2:**
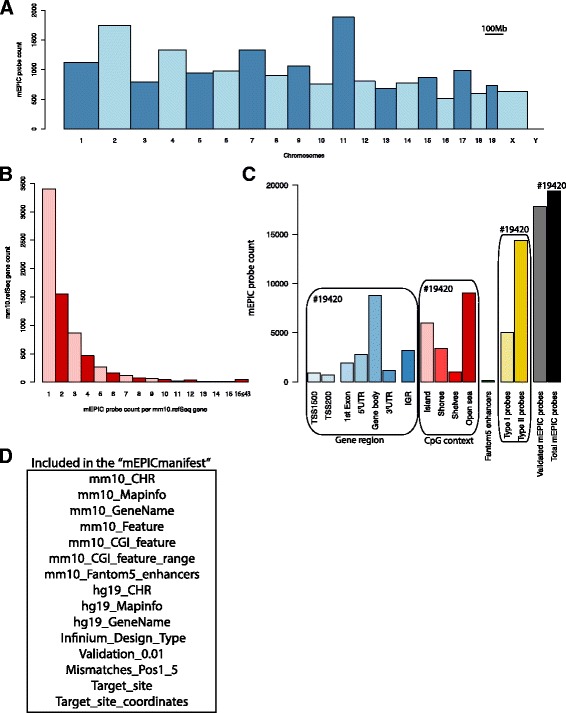
Genomic distribution and annotation of mEPIC probes. **a** Genomic distribution of mEPIC probes across mouse chromosomes. Bar width illustrates chromosome size. **b** Barplot illustrating that only few mEPIC probes map per mm10.refSeq gene. 16 ≤ 43: counts number of annotated mm10.refSeq genes with ≥16 and ≤43 mEPIC probes. **c** Barplot of genomic features (Gene region, CpG context and FANTOM5 enhancers), probe design (type I and II), validated (detection *P*-value < 0.01) and total mEPIC probes. Rounded rectangles enclose features, which add up to the total number of identified mEPIC probes (#19,420) **d** Overview of annotations included in the “mEPICmanifest” in Additional file 2. Mm10: *Mus musculus* genome build 10, CHR: Chromosome, CGI: CpG island

Genomic locations revealed that mEPIC probes were well distributed between chromosomes with a maximum number of probes (1886) located in chromosome 11 and a minimum number of probes (513) in chromosome 16 (Fig. 2a). To annotate the 19,420 identified mEPIC probes, RefSeq annotated genes were subsequently downloaded from the UCSC genome table knownGenes (mm10.refGene) and complemented with 1500 nucleotides upstream from the transcriptional start position to include proximal promoters.

Overlap analysis revealed that approximately 84% (16,352 out of 19,420) of mEPIC probes overlapped with annotated mm10.refSeq genes, but that each gene was targeted by only a few probes (Fig. 2b), therefore limiting the use of EPIC for certain applications such as detection of differentially methylated regions (DMRs). Nevertheless, the utility of EPIC array for DNA methylation analysis using mouse samples remains suitable for broader applications such as cluster analysis.

Comparison of mouse and human gene names for individual mEPIC probes revealed that a large fraction (> 80%) were common between the two species, thus suggesting that genomic regions covered by mEPIC probes are highly conserved between humans and mice. Considering that high sequence homology generally occurs within exonic regions we hypothesized that mEPIC probes primarily map to exons. To test this, we examined mEPIC probes in the light of RefSeq transcript information (exon start and end positions) extracted from the UCSC genome table browser. Overlap analysis between mEPIC probes and mm10.RefSeq exons revealed that 72% of gene-associated mEPIC probes (11,732 out of 16,352) were indeed located in exons, corresponding to an overall 60% of the total 19,420 mEPIC probes.

We subsequently examined gene- and CpG-related features of the mEPIC probes (Fig. 2c) as conventionally used and provided for the human EPIC manifest [11]. Overlap analysis revealed that all genomic features, including TSS1500 (200–1500 bases upstream from the transcriptional start site, TSS), TSS200 (0–200 bases upstream from the TSS), 1st Exon, 5’UTR (5′ untranslated region), gene body, 3’UTR (3′ untranslated region), IGR (intergenic region) and FANTOM5 enhancers, were represented. However, the majority of mEPIC probes (8756) were located in gene bodies. Annotations of features related to CpG context, i.e. CpG islands (CGIs), shores, shelves and open seas revealed a predominant representation of mEPIC probes in open seas (8756). Finally, we determined that mouse FANTOM5 enhancers were targeted to the least extent (112) (Fig. 2c). Hence the Infinium MethylationEPIC beadchips coverage of human FANTOM5 enhancers (captured by 350,000 sites) [7] does not translate to the mouse genome, which is consistent with previous studies reporting that enhancers tend to evolve faster than coding regions due to mechanisms such as enhancer deletion, alterations in transcription factor binding sites and/or acquisition of new enhancers [12].

Annotations of mEPIC probes are summarized in Fig. 2c, d and described in detail in Additional file 2, referred to as the “mEPICmanifest”.

### Experimental validation of mEPIC probes

Finally, we aimed to validate mEPIC probes experimentally by performing DNA methylation analysis of mouse samples using Infinium MethylationEPIC BeadChips. To reduce sample heterogeneity as a possible bias for DNA methylation analysis we used genomic DNA extracted from sorted myeloid cells with >95% purity of C57BL/6 mice. Six samples arising from 3 cell types in biological duplicates were hybridized on two slides. Idat files were processed through scripts adapted from the ChAMP Bioconductor Package [13] which filters probes based on a defined detectable *P*-value cut-off.

**Fig. 3:**
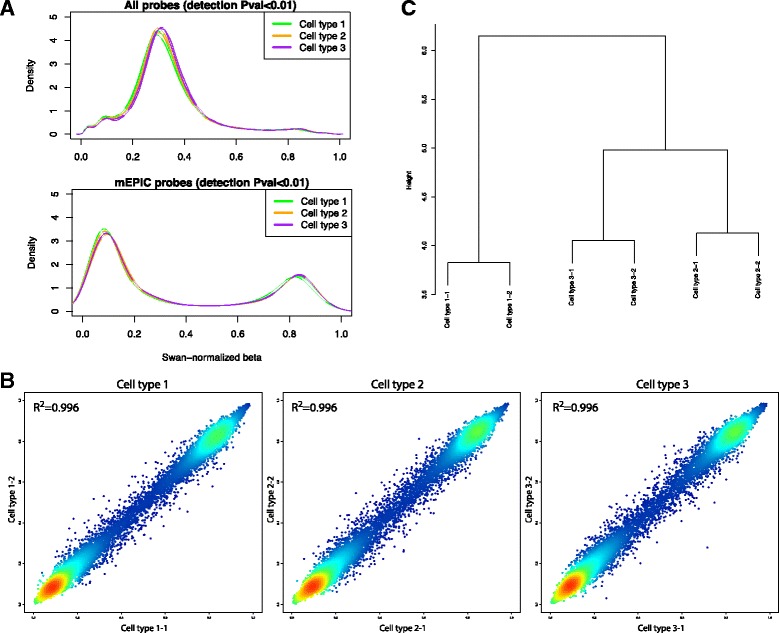
Experimental validation of mEPIC probes. **a** Density plots of SWAN-normalized DNA methylation values from all probes (upper panel) or mEPIC probes only (lower panel) passing detection *P*-value of 0.01. **b** Scatter density plot of SWAN-normalized mEPIC DNA methylation values comparing biological duplicates of cell types 1, 2 and 3, respectively. **c** Hierarchical clustering of SWAN-normalized mEPIC probes passing detection *P*-value of 0.01

As a first unbiased approach we examined the detection signal of all EPIC probes and identified a total of 263,029 that passed the detection *P*-value cut-off of 0.01 (default settings), thus contrasting with the 19,420 mEPIC probes identified in silico. This discrepancy might result from ambiguous hybridization or, on the contrary, might suggest that additional signals could be used for DNA methylation studies in mouse samples. We first addressed this issue in silico by loosening the mapping criteria of EPIC probes and applying a higher mismatch allowance within the Bismark alignment. However, the number of uniquely mapped hits peaked at only 20,337 when 2 mismatches were permitted, as additional mismatch allowance resulted in ambiguous mapping, i.e. mapping of probes to multiple target regions. We then addressed whether a shorter part of the EPIC probe could be responsible for hybridization and subsequent generation of DNA methylation signals. Reduction of the length of the probe sequence to 20 nucleotides in silico and subsequent mapping to the mouse genome resulted in marked decrease in uniquely mapped hits, concomitant with an increase in ambiguous hits. We next examined the DNA methylation density distribution and observed that the 263,029 probes passing the detection *P*-value cut-off peaked at 0.3 (Fig. 3a), which has previously been described as a failed experimental signal [5]. In contrast, when only mEPIC probes (passing a detection *P*-value threshold of 0.01) were considered, the DNA methylation values displayed an expected bimodal distribution peaking at low (~0.1) and high (~0.85) beta values (Fig. 3a).

Due to pooled samples with mixed gender, 633 mEPIC probes mapping to chromosome X (none of the mEPIC probes mapped to chromosome Y), as well as 31 mEPIC probes known to target non-CpG sites in humans, had been filtered prior to the analysis. Of the 18,756 remaining mEPIC probes, 18,559 (~99%) passed the detection *P*-value threshold of 0.01 and were subsequently listed in the “mEPICmanifest” as “Validation_0.01” (Additional file 2). Of notice, probes passing the detection *P*-value cut-off could still represent a bias due to putative disruption of the CpG target site. We therefore examined the sequence of mEPIC target sites in greater detail and found that, as expected, the majority of mEPIC probes (72%) did contain a CpG dinucleotide. The remaining probes predominantly targeted non-CpG (or CH) sites (13%), i.e. CpA (9%), CpT (2%) or CpC (2%) and TpG dinucleotides (9%) of which the latter could result from C > T transitions, a common DNA methylation-mediated mutation caused by deamination of 5-methylcytosine [14]. Moreover, mismatches between EPIC probes and genomic DNA can compromise hybridization and thereby also influence estimation of DNA methylation levels. Knowing that internal single nucleotide polymorphisms (SNPs) at more than 5 bases from the 3’end of EPIC probes have negligible effect [15] we predominantly focused on probe positions 1–5 (probes had been reverse/complemented prior to mapping). After retrieving mismatch information from Bismark-derived BAM files a total of 2954 mismatches were identified between mEPIC probes and the mm10 reference genome at probe positions 1–5. Target site sequence context and mismatch information have been included in columns “Target_site” and “Mismatches_Pos1_5” of the mEPICmanifest (Additional file 2).

We aimed to further examine whether within array normalization, for example using Subset-quantile within array normalization (SWAN) [16] or BMIQ [17] could be affected by the restricted number of mEPIC probes. To this end, human EPIC data was loaded, filtered, and normalized in the same manner as the mouse data (mEPIC processed) and subsequently compared to data where mEPIC probes were filtered post-normalization (“conventionally” processed). mEPIC and “conventionally” processed human data showed a correlation of R^2^ > 0.999 (Additional file 3) suggesting that within array normalization is not affected by the limited number of mEPIC probes. Standard tools for analysis of human Infinium MethylationEPIC BeadChip data therefore seem applicable to mEPIC data.

Reproducibility of DNA methylation signals between replicates is an important parameter for successful DNA methylation studies. To assess this we compared DNA methylation of biological replicates run on different slides for which we detected high correlations as reflected by a R^2^ value of 0.996 (Fig. 3b). Finally, we demonstrated the utility of mEPIC probes for data exploration. Hierarchical clustering revealed that biological replicates cluster together according to cell type. Since all cell types are of myeloid origin, this indicates that even small biological differences can be readily detected using mEPIC probes.

### Mapping of EPIC probes to additional animal models and mouse strains

**Fig. 4:**
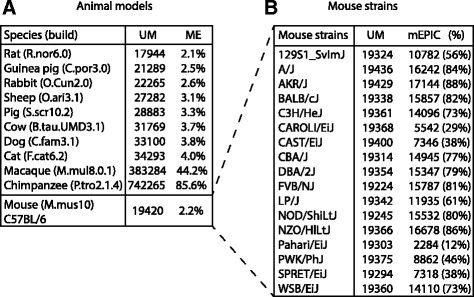
Mapping of EPIC probes to commonly used animal models and different mouse strains. Alignment results of EPIC probes mapped to **a** Commonly used animal models and **b** mouse strains. The “mEPIC” column contains the number (and percentage) of probes represented in the mEPICmanifest, i.e. overlapping with C57BL/6. Alignment was conducted with default settings: mismatch allowance (−n) = 1 and seed length (−l) = 28) with reporting of Uniquely Mapped hits (UM) and Mapping Efficiency (ME), respectively

Given the successful demonstration of Infinium MethylationEPIC BeadChips for mouse samples we sought to examine mappability of EPIC probes to additional commonly used laboratory animals such as Rat, Guinea pig, Rabbit, Sheep, Pig, Cow, Dog, Cat, Macaque and Chimpanzee (Fig. 4a). The number of uniquely mapped hits for rodents (Rat: 17,944, Guinea pig: 21,289 and Rabbit: 22,265) was as expected comparable with Mouse (19,420), while species genetically closer to humans showed higher mappability with a maximum of 742,265 potential probes for Chimpanzee. Mapping of EPIC probes to genetically more distant animals such as Chicken, Zebrafish and Fruit fly resulted in a low number of uniquely mapped hits (2446, 347, 35, respectively) and were therefore not reported in further detail. For each species EPIC probes with uniquely mapped hits, genomic locations (chromosome, mapinfo and strand information) and Infinium design type were listed in Additional files 4, 5, 6, 7, 8, 9, 10, 11, 12, 13.

Lastly, we examined whether different mouse strains (listed in Fig. 4b) would give a similar coverage as C57BL/6 (mm10/GRCm38) by mapping EPIC probes to genomes available from the Mouse Genomes Project. All mouse strains (total of 17) showed a similar number of uniquely mapped hits (range 19,245–19,436) (Fig. 4b). However, comparison of these with mEPIC probes (i.e. overlapping with C57BL/6 (mm10/GRCm38)) revealed variation amongst strains ranging from 2284 (Pahari/EiJ) to 17,144 (AKR/J). Hence, the mEPICmanifest is not equally useful for all mouse strains. In order to facilitate the application of DNA methylation analysis using EPIC for other mouse strains we listed EPIC probes with uniquely mapped hits, genomic locations (chromosome, mapinfo and strand information) and Infinium design type in Additional files 14, 15, 16, 17, 18, 19, 20, 21, 22, 23, 24, 25, 26, 27, 28, 29, 30.

## Conclusions

We herein demonstrate the potential usability of Illumina Infinium MethylationEPIC BeadChips for mouse samples. Using in silico analysis we identified 19,420 mEPIC probes of which 18,559 were experimentally validated and compared between inter-array biological replicates, thereby demonstrating reliable and reproducible results. Annotation analysis of mEPIC probes applied to the mm10 mouse genome revealed that mEPIC probes were distributed throughout the genome, predominantly covered annotated RefSeq genes and encountered similar genomic features (with reduced coverage), as the human counterpart. A summary of mEPIC probe characteristics is listed in the “mEPICmanifest” available in Additional file 2. The mapping analysis was subsequently applied to 17 additional mouse strains and 10 commonly used animal models with mapping info made available in Additional files 14, 15, 16, 17, 18, 19, 20, 21, 22, 23, 24, 25, 26, 27, 28, 29, 30. Finally, we give an example of DNA methylation analysis (e.g. hierarchical clustering) that can be conducted with mouse samples.

In conclusion, our study demonstrates that human Infinium MethylationEPIC BeadChip array is a valid and affordable platform for studying DNA methylation in mouse samples.

## Methods

### Mouse samples

All C57BL/6 mice were bred and maintained under specific pathogen-free conditions in the animal facility at Karolinska University Hospital (Stockholm, Sweden). Extraction of genomic DNA was performed using QIAamp DNA Micro Kit (Qiagen). Three cell populations were isolated from mouse bone marrow using: Monocyte isolation kit (Miltenyi Biotec) for cell type 1 and sorting of Lin^−^ckit^+^sca1^−^CD34^+^CD16/32^int^ or Lin^−^ckit^+^CD115^+^Ly6C^+^CD11b^−^ populations using a BD influx sorter for cell type 2 and 3, respectively.

### Mapping of EPIC probes

EPIC probes were downloaded from the Illumina website (http://support.illumina.com/array/array_kits/infinium-methylationepic-beadchip-kit/downloads.html), converted to fasta format and processed to their reverse/complements. Processed EPIC probes are available in Additional file 1 in fasta format. Mouse (mm10) and human (hg19) genomes were downloaded from Ensembl (http://www.ensembl.org/info/data/ftp/index.html/), whereas different mouse strain genomes were downloaded from the Mouse Genomes Project (ftp://ftp-mouse.sanger.ac.uk/REL-1504-Assembly/). The “bismark_genome_preparation” function of Bismark (version 0.14.5) [8] with default parameters, was used for in silico bisulfite conversion of respective reference genomes (i.e. bismark_genome_preparation --bowtie1 –verbose <path_to_genome_folder>). Subsequently, the “bismark” function, which we set to rely on Bowtie 1 (version 1.1.2) [10] was used for mapping of EPIC probes (i.e. bismark --bowtie1 -n 1 -l 28 < path_to_genome_folder > −f EPICprobes.fa –o < path_to_output_directory>).

### Mismatch detection

Mismatch information was extracted from Bismark-derived BAM files using the calmd –e function of Samtools (version 1.5) [18]. Since Bismark-derived MD tags also include in silico C > T conversions, “true mismatches” were identified as discrepancies between mismatch positions reported in MD tags and C > T conversions reported as “x”, “h” or “z” in MZ tags. Mismatches detected at positions 1–5 of reverse/complemented mEPIC probe, were subsequently flagged in column “Mismatches_Pos1_5” of the mEPICmanifest.

### Annotation of mEPIC probes

RefSeq genes were downloaded from UCSC genome table knownGenes (mm10.refGene) (https://genome.ucsc.edu/cgi-bin/hgTables). 1500 nucleotides were subsequently added to the “txStart” of each transcript to ensure inclusion of proximal promoters in the annotation. Overlap analyses between mEPIC probes and RefSeq transcripts/exons were conducted with the “intersect” function of BEDTools (version 2.25.0) [19]. Count of mEPIC probes covering RefSeq transcripts and probe number per transcript was conducted with in house bash scripts. Overlap analysis of mEPIC probes with genomic features such as TSS1500, TSS200, 1st Exon, 5’UTR, Gene body, 3’UTR and IGR was conducted as previously suggested [11]. Noticeably, when multiple transcripts overlapped with the same CpG site, the following priority was given: TS200 > TSS1500 > 5’UTR > 1st Exon > Body >3’UTR > IGR. Overlap of mEPIC probes with CpG islands, Shores, Shelves or Open Sea was determined using the “annotatr” (version v1.1.3) Bioconductor package [20]. FANTOM5 enhancers were downloaded from http://fantom.gsc.riken.jp/5/datafiles/latest/extra/Enhancers/ and overlap with mEPIC probes was determined with the intersect function of BEDTools (version 2.25.0) [19]. Information of type I and II probes was retrieved from the EPIC manifest available from the Illumina website (http://support.illumina.com/array/array_kits/infinium-methylationepic-beadchip-kit/downloads.html). Target site sequence context was retrieved via http://togows.org/api/ucsc/mm10/TargetSite_GenomicCoordinates.fasta.

### DNA methylation analysis

The Infinium Human MethylationEPIC BeadChip (Illumina) platform was used for DNA Methylation profiling. Samples were randomized on 2 slides and processed by the core facility for Bioinformatics and Expression Analysis (BEA), Karolinska Institutet, Huddinge campus. Idat files were loaded into R using scripts adapted from the ChAMP package (version 2.6.0) [13] referred to as “mEPIC.loading.Script.R” and “Champ.load.mEPIC.Script.R” available in Additional files 31 and 32, respectively. Furthermore, an R script for normalization of mEPIC probes with Subset-quantile within array normalization (SWAN) [16] and BMIQ [21], referred to as “Champ.norm.mEPIC.Script.R” is available in Additional file 33. Hierarchical clustering was performed with the ward.D2 method of the hclust function in R.

## Additional files

Additional file 1: EPICprobes. (TXT 54191 kb)

Additional file 2: mEPICmanifest. (XLS 5446 kb)

Additional file 3: Figure S1. Human_EPIC. (PDF 692 kb)

Additional file 4: Rat_EPIC. (XLS 1680 kb)

Additional file 5: GuineaPig_EPIC. (XLS 1914 kb)

Additional file 6: Rabbit_EPIC. (XLS 2083 kb)

Additional file 7: Sheep_EPIC. (XLS 2552 kb)

Additional file 8: Pig_EPIC. (XLS 2701 kb)

Additional file 9: Cow_EPIC. (XLS 2971 kb)

Additional file 10: Dog_EPIC. (XLS 3095 kb)

Additional file 11: Cat_EPIC. (XLS 3076 kb)

Additional file 12: Macaque_EPIC. (XLS 6450 kb)

Additional file 13: Chimpanzee_EPIC. (XLS 6473 kb)

Additional file 14: 129S1_SvImJ_EPIC. (XLS 1809 kb)

Additional file 15: A_J_EPIC. (XLS 1819 kb)

Additional file 16: AKR_J_EPIC. (XLS 1818 kb)

Additional file 17: BALB_cJ_EPIC. (XLS 1810 kb)

Additional file 18: C3H_HeJ_EPIC. (XLS 1812 kb)

Additional file 19: CAROLI_EiJ_EPIC. (XLS 1813 kb)

Additional file 20: CAST_EiJ_EPIC. (XLS 1816 kb)

Additional file 21: CBA_J_EPIC. (XLS 1808 kb)

Additional file 22: DBA_2J_EPIC. (XLS 1811 kb)

Additional file 23: FVB_NJ_EPIC. (XLS 1799 kb)

Additional file 24: LP_J_EPIC. (XLS 1810 kb)

Additional file 25: NOD_ShiLtJ_EPIC. (XLS 1801 kb)

Additional file 26: NZO_HlLtJ_EPIC. (XLS 1812 kb)

Additional file 27: Pahari_EiJ_EPIC. (XLS 1807 kb)

Additional file 28: PWK_PhJ_EPIC. (XLS 1813 kb)

Additional file 29: SPRET_EiJ_EPIC. (XLS 1806 kb)

Additional file 30: WSB_EiJ_EPIC. (XLS 1812 kb)

Additional file 31: mEPIC.loading.Script.R. (R 2 kb)

Additional file 32: Champ.load.mEPIC.Script.R. (R 6 kb)

Additional file 33: Champ.norm.mEPIC.Script.R. (R 10 kb)

## Abbreviations

3′UTRs: 3′ untranslated region
5′UTR: 5′ untranslated region
A: Adenosine
BS: (sodium) bisulfite
C: Cytosine
CGI: CpG island
DMR: Differentially methylated region
DNMT: DNA methyltransferase
HM27: Infinium HumanMethylation27 BeadChip array
HM450: Infinium HumanMethylation450 BeadChip array
IGR: Intergenic region
-l: Seed length
mEPIC: Mouse EPIC
-n: Mismatch allowance
SNP: Single Nucleotide Polymorphism
SWAN: Subset-quantile within array normalization
T: Thymine
TSS: Transcription start site
TSS1500: 200–1500 bases upstream from the TSS
TSS200: 0–200 bases upstream from the TSS
UTR: untranslated region

## References

[1] Li E, Zhang Y (2014). DNA methylation in mammals. Cold Spring Harb Perspect Biol.

[2] Kouzarides T (2007). Chromatin modifications and their function. Cell.

[3] Liu Y, Aryee MJ, Padyukov L, Fallin MD, Hesselberg E, Runarsson A, Reinius L, Acevedo N, Taub M, Ronninger M (2013). Epigenome-wide association data implicate DNA methylation as an intermediary of genetic risk in rheumatoid arthritis. Nat Biotechnol.

[4] Joehanes R, Just AC, Marioni RE, Pilling LC, Reynolds LM, Mandaviya PR, Guan W, Xu T, Elks CE, Aslibekyan S (2016). Epigenetic signatures of cigarette smoking. Circulation Cardiovascular Genetics.

[5] Wong NC, Ng J, Hall NE, Lunke S, Salmanidis M, Brumatti G, Ekert PG, Craig JM, Saffery R (2013). Exploring the utility of human DNA methylation arrays for profiling mouse genomic DNA. Genomics.

[6] Chopra P, Papale LA, White AT, Hatch A, Brown RM, Garthwaite MA, Roseboom PH, Golos TG, Warren ST, Alisch RS (2014). Array-based assay detects genome-wide 5-mC and 5-hmC in the brains of humans, non-human primates, and mice. BMC Genomics.

[7] Pidsley R, Zotenko E, Peters TJ, Lawrence MG, Risbridger GP, Molloy P, Van Djik S, Muhlhausler B, Stirzaker C, Clark SJ (2016). Critical evaluation of the Illumina MethylationEPIC BeadChip microarray for whole-genome DNA methylation profiling. Genome Biol.

[8] Krueger F, Andrews SR (2011). Bismark: a flexible aligner and methylation caller for bisulfite-Seq applications. Bioinformatics.

[9] Xi Y, Li W (2009). BSMAP: whole genome bisulfite sequence MAPping program. BMC bioinformatics.

[10] Langmead B, Trapnell C, Pop M, Salzberg SL (2009). Ultrafast and memory-efficient alignment of short DNA sequences to the human genome. Genome Biol.

[11] Moran S, Arribas C, Esteller M (2016). Validation of a DNA methylation microarray for 850,000 CpG sites of the human genome enriched in enhancer sequences. Epigenomics.

[12] Rubinstein M, de Souza FS (2013). Evolution of transcriptional enhancers and animal diversity. Philos Trans R Soc Lond Ser B Biol Sci.

[13] Morris TJ, Butcher LM, Feber A, Teschendorff AE, Chakravarthy AR, Wojdacz TK, Beck S (2014). ChAMP: 450k Chip analysis methylation pipeline. Bioinformatics.

[14] Cooper DN, Krawczak M (1989). Cytosine methylation and the fate of CpG dinucleotides in vertebrate genomes. Hum Genet.

[15] Zhou W, Laird PW, Shen H (2017). Comprehensive characterization, annotation and innovative use of Infinium DNA methylation BeadChip probes. Nucleic Acids Res.

[16] Maksimovic J, Gordon L, Oshlack A (2012). SWAN: subset-quantile within array normalization for illumina infinium HumanMethylation450 BeadChips. Genome Biol.

[17] Teschendorff AE, Marabita F, Lechner M, Bartlett T, Tegner J, Gomez-Cabrero D, Beck S (2013). A beta-mixture quantile normalization method for correcting probe design bias in Illumina Infinium 450 k DNA methylation data. Bioinformatics.

[18] Li H, Handsaker B, Wysoker A, Fennell T, Ruan J, Homer N, Marth G, Abecasis G, Durbin R (2009). Genome project data processing S: the sequence alignment/map format and SAMtools. Bioinformatics.

[19] Quinlan AR, Hall IM (2010). BEDTools: a flexible suite of utilities for comparing genomic features. Bioinformatics.

[20] Cavalcante RG, Sartor MA (2017). Annotatr: genomic regions in context. Bioinformatics.

[21] Marabita F, Almgren M, Lindholm ME, Ruhrmann S, Fagerstrom-Billai F, Jagodic M, Sundberg CJ, Ekstrom TJ, Teschendorff AE, Tegner J (2013). An evaluation of analysis pipelines for DNA methylation profiling using the Illumina HumanMethylation450 BeadChip platform. Epigenetics.

