# Supplementary material for: Usability of human Infinium MethylationEPIC BeadChip for mouse DNA methylation studies
Source: BMC Bioinformatics. 2017 Nov 15;18:486. doi: 10.1186/s12859-017-1870-y (PMC5688710; doi:10.1186/s12859-017-1870-y)
Supplement: Supplementary file 3 — Human_EPIC. (PDF 692 kb) [file 12859_2017_1870_MOESM3_ESM.pdf]

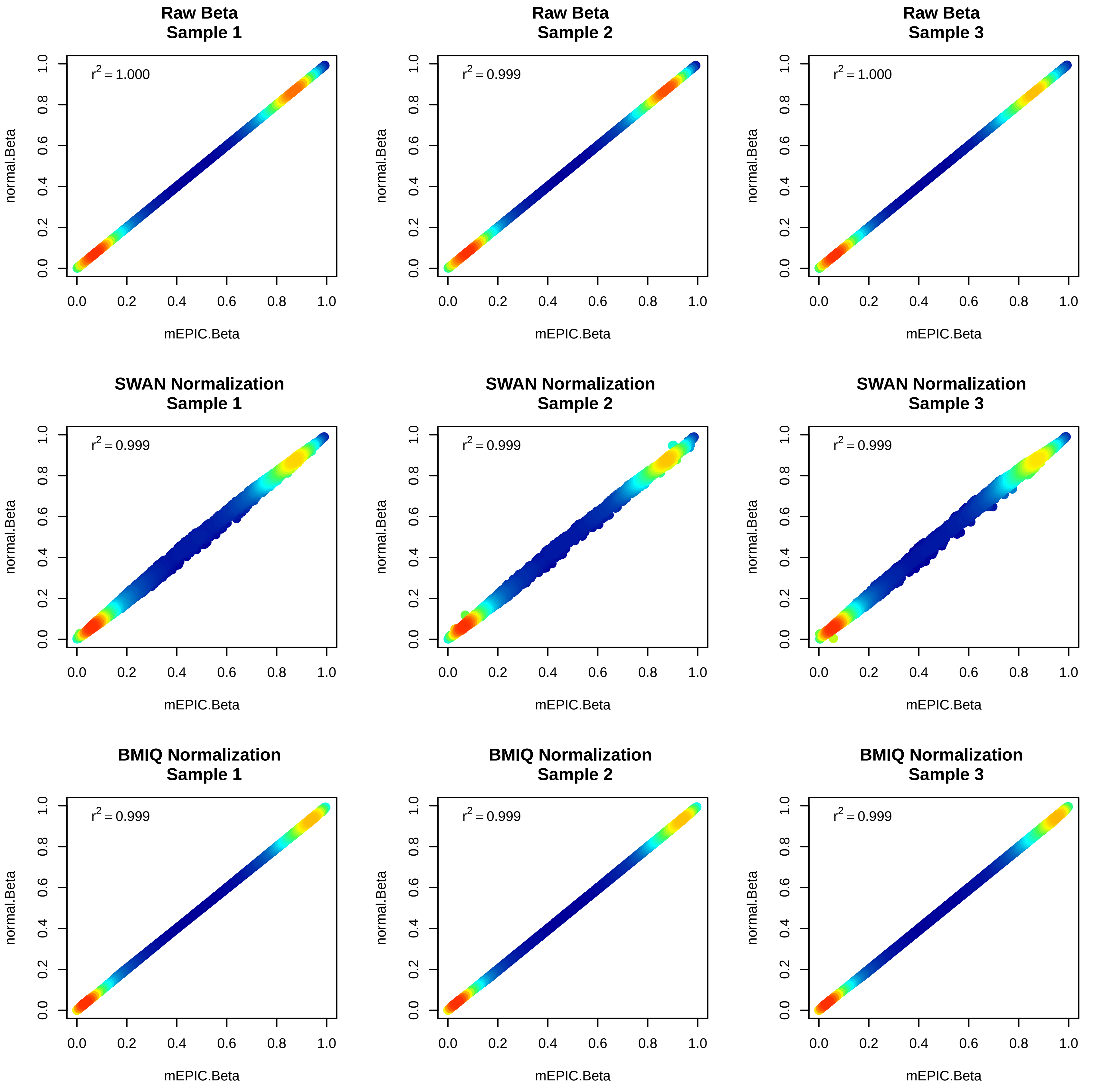
Raw Beta Sample 1
Raw Beta Sample 2
Raw Beta Sample 3
SWAN Normalization Sample 1
SWAN Normalization Sample 2
SWAN Normalization Sample 3
BMIQ Normalization Sample 1
BMIQ Normalization Sample 2
BMIQ Normalization Sample 3
r2 = 1.000
r2 = 0.999
r2 = 1.000
r2 = 0.999
r2 = 0.999
r2 = 0.999
r2 = 0.999
r2 = 0.999
r2 = 0.999
normal.Beta
mEPIC.Beta
0.0
0.2
0.4
0.6
0.8
1.0
